# Strichnine Locally Applied

**Published:** 1835

**Authors:** 


IV.—*Strichnine Locally Applied in Paralysis.*—In the N. A. Archives, for March of the present year, Professor Smith, of Baltimore, has given the following case:—

"Two years since, a seaman was received into the Baltimore Infirmary, laboring under paralysis of the exterior muscles of the left leg. He stated that, some days before, he had received a blow on the anterior part of the thigh, which had produced contusion and consequent soreness of the muscles. The immediate effects of the injury soon passed away, but there supervened an increasing sense of weakness in the member, which soon terminated in paralysis. The muscles on the anterior part of the thigh now felt soft, relaxed, and flabby. When the patient carefully kept the leg perfectly extended upon the thigh, he was able to bear his weight upon the member; but whenever he flexed it in the slightest degree, in attempting to walk, the leg was instantly doubled under him, and, if he trusted to it for support, he fell. When he was seated in a high chair, and the leg allowed to hang downward, he had no power to swing it forward.

"This man had been laboring under some slight gastric disorder which appropriate remedies had relieved. I could detect no evidence of spinal irritation. The local affection appeared to exist alone. Stimulating frictions were resorted to with but little effect. The cold douche was also tried with as little benefit. I then directed a small blister to be applied upon the anterior part of the thigh, near its middle. When vesication had been produced, the cuticle was removed, and one fourth of a grain of strichnine applied to the denuded surface. This was done in the evening. In the course of that night the patient was waked by involuntary spasmodic twitches of the diseased muscles, occurring at frequent intervals, and rendering it impossible for him to keep the limb quiet in bed. This continued at intervals during the night. In the morning, when the patient rose, he found, to his surprise, that he had now the power of extending the leg upon the thigh, and of securely standing and walking, although the member had not recovered all its natural power. On the following evening I directed the application to be repeated. The same involuntary movements did not recur, but further improvement took place in the contractile power of the muscles. This improvement, without further resort to the remedy, continued to progress, and in a few days the natural strength of the member was perfectly restored.

"The result of this case has induced me, in several cases of local palsy, to resort to the same remedy, but I have by no means been equally successful in such. In one case, however, of paralysis of the left arm and fore-arm, partial success was obtained. The same involuntary twitches of the muscles took place in this case, attended with some pain." p. 386–387.

